# Case Report: A Novel *GNB1* Mutation Causes Global Developmental Delay With Intellectual Disability and Behavioral Disorders

**DOI:** 10.3389/fneur.2021.735549

**Published:** 2021-09-27

**Authors:** Jorge Diogo Da Silva, Marta Daniela Costa, Bruno Almeida, Fátima Lopes, Patrícia Maciel, Andreia Teixeira-Castro

**Affiliations:** ^1^Life and Health Sciences Research Institute (ICVS), School of Medicine, University of Minho, Braga, Portugal; ^2^ICVS/3B's—Portuguese Government Associate Laboratory, Braga, Guimarães, Portugal; ^3^Pediatrics Department, Hospital of Santa Maria Maior, Barcelos, Portugal

**Keywords:** neurodevelopment, G proteins, intellectual disability, *GNB1*, neurogenetics

## Abstract

Diseases of neurodevelopment mostly exhibit neurological and psychiatric symptoms that go from very mild to extremely severe. While the etiology of most cases of neurodevelopmental disease is still unknown, the discovery of underlying genetic causes is rapidly increasing, with hundreds of genes being currently implicated as disease-causing. Here, we report a clinical case of a patient with a previously undiagnosed syndrome comprising severe global developmental delay, intellectual disability, and behavioral disorders (such as attention-deficit/hyperactivity disorder, autism spectrum disorder and recurrent bouts of aggressive behavior). After genetic testing, a pathogenic variant was detected in the *GNB1* gene, which codes for the G-protein subunit β1. The detected variant (c.217G>A, p.A73T) has not been previously reported in any of the 58 published cases of GNB1 encephalopathy. However, it localizes to the mutational hotspot in exons 6 and 7 in which 88% of all missense mutations occur. An *in silico* model predicts that this mutation is likely to disrupt the WD40 domain of the GNB1 protein, which is required for its interaction with other G-proteins and, consequently, for downstream signal transduction. In conclusion, we reported an additional GNB1 encephalopathy patient, bearing a novel mutation, taking another step toward a better understanding of its clinical presentation and prospective development of treatments for the disease.

## Introduction

Knowledge on the genetic causes of neurodevelopmental diseases has been increasingly growing. Among the hundreds of genes that have been established as neurodevelopmental disease-causing, genes that encode for guanine nucleotide-binding proteins (G proteins) have recently been included (1, 2). Specifically, mutations in *GNB1*, which encodes for the G protein subunit β1, have been recently described as causal of a neurodevelopmental syndrome (1, 3). The Gβ subunit forms a heterotrimer with Gα and Gγ in the steady-state which is bound to a membrane G protein-coupled receptor (GPCR) (4). Upon binding of a specific extracellular ligand, there is separation of the Gα from the Gβγ dimer, and consequently activation of an intracellular signaling cascade (4). This is a ubiquitous signaling pathway, which is in line with the fact that its components, including *GNB1*, are ubiquitously expressed (5). In neurons, *GNB1* is also widely expressed in all brain regions, but enriched in rod photoreceptor cells of the retina (5).

Pathogenic variants in *GNB1* cause a heterogeneous neurodevelopmental syndrome named GNB1 encephalopathy (OMIM: 616973) whose unifying characteristic is a global development delay (GDD), present in 100% of patients (3, 6). Manifestations that are present in at least 50% of patients include moderate-to-severe intellectual disability (ID), abnormal muscle tone, abnormal vision, epilepsy, and gastrointestinal abnormalities (3, 6). Several other neuropsychiatric and non-neuropsychiatric symptoms have also been reported, albeit less frequently (3, 6). Nevertheless, clinical data is very limited since only 58 patients with clinical-impacting *GNB1* variants have been reported (1, 3, 7–19). Moreover, this syndrome can easily overlap with many conditions, being clinically indistinguishable from other neurodevelopmental disorders.

An interesting finding is that, out of the 51 patients with pathogenic missense variants, 25 (49%) are located in exon 6, and 20 (39%) are in exon 7, indicating the presence of a mutational hotspot (1, 3, 7–19). Of note, 13 patients (25%) bear a p.(Ile80Thr) variant, which further supports such hypothesis (Table 1). As previously suggested, these mutations are likely to affect residues from the interacting interface of the GNB1 protein, compromising its binding to the other G protein subunits (9). Here, we describe a Portuguese patient with GDD bearing the first pathogenic *GNB1* missense mutation affecting residue alanine 73 (A73), provide an *in silico* prediction of how it affects protein function and integrate the clinical findings with the previously described cases.

**Table 1:** List of all patients with clinically relevant *GNB1* mutations currently published.

**Affected exon**	**Mutation type**	**Mutation**	**Total** **patient no**.	**Reference**														
				**1**	**3**	**7**	**8**	**9**	**10**	**11**	**12**	**13**	**14**	**15**	**16**	**17**	**18**	**19**
5	Missense	p.Arg52Gly	1										1					
	Missense	p.Gly53Glu	2		1											1		
	Missense	p.Gly64Val	1										1					
6	Missense	p.Ser74Leu	1											1				
	Missense	p.Asp76Gly	1	1														
	Missense	p.Asp76Glu	1	1														
	Missense	p.Gly77Ser	2	1													1	
	Missense	p.Gly77Ala	1			1												
	Missense	p.Gly77Val	1					1										
	Missense	p.Gly77Arg	1		1													
	Missense	p.Lys78Arg	2	1	1													
	Missense	p.Ile80Asn	2	2														
	Missense	p.Ile80Thr	13	3	8		1							1				
	Missense	p.Lys89Arg	1		1													
	Splice Site	c.268-1 G>T	1										1					
7	Deletion	p.His91Profs*9	1										1					
	Missense	p.Ala92Thr	1										1					
	Missense	p.Ala92Asp	1		1													
	Missense	p.Pro94Ser	1										1					
	Missense	p.Leu95Pro	5	1	2					1				1				
	Missense	p.Arg96Leu	3										3					
	Missense	p.Met101Val	2	2														
	Missense	p.Ala106Thr	1										1					
	Missense	p.Cys114Tyr	1		1													
	Missense	p.Asp118Gly	3		2													1
	Missense	p.Asp118Tyr	1									1						
9	Nonsense	p.Trp211*	1												1			
10	Splice Site	c.700-1 G>T	1						1									
	Missense	p.Ser279Phe	1								1							
	Deletion	p.Gly306Cysfs*4	1										1					
11	Splice Site	c.917-1 G>T	1										1					
	Missense	p.Ala326Thr	1	1														
	Deletion	p.Gly330Valfs*4	1						1									
		**Total**	58															

## Case Report

A 15 year 8-month old female patient was born at 36 weeks gestation with a weight of 2880 g (P21), length of 47 cm (P12), and head circumference of 33 cm (P23). Pregnancy was uncomplicated, and birth was by cesarean section due to prolonged labor, but otherwise unremarkable, with normal Apgar scores. Metabolic screening and otoacoustic emissions were normal. Parents (mother with 30 and father with 33 years of age) were non-consanguineous and healthy, and the patient has a healthy younger sibling. Regarding developmental milestones, the patient showed a social smile at 2 months, sat unsupported at 9 months, walked autonomously at 17 months, spoke the first intelligible words at 4 years and built sentences at 5 years of age, indicating a global developmental delay (GDD), which was further supported by the Griffiths III Mental Development Scale assessment (general quotient of 52 at 9 years of age). Pubertal development is adequate for the current age.

In addition to GDD, the patient fulfilled clinical criteria for autism spectrum disorder (ASD) at 18 months of age, due to impaired social interaction, restricted interests, and repetitive behavior. Throughout development, the patient has additionally met the criteria for other behavioral disorders, namely attention-deficit/hyperactivity disorder (ADHD) and oppositional defiant disorder. Moreover, the patient was diagnosed with mild-to-moderate intellectual disability (IQ of 52 as measured by the WISC-III). Finally, the patient met the criteria for a mixed language disorder, as well as developmental coordination disorder. No other specific neurological symptoms were observed, namely dystonia (or other movement disorders), sensorineural hearing loss, visual symptoms or epilepsy. Furthermore, no structural abnormalities were observed in two brain MRIs (one at 3 years and another at 15 years of age), nor electroencephalographic changes (studied at 18 months of age). No dysmorphic features were observed.

At the current age of 15, the most impacting symptoms are related with behavior, with the patient showing repeated bouts of auto and hetero-aggressive behavior, attention deficit and a frequently obsessive conduct. Multiple treatment regimens have been tried, including several drug types, namely antipsychotics, antidepressants, mood stabilizers and stimulants, albeit with mixed results in the management of the psychiatric disorder. Multiple episodes of extrapyramidal adverse effects of antipsychotics have also been observed in the patient.

Considering the clinical presentation of the patient, genetic testing was undertaken. A karyotype was normal, and a comparative genomic hybridization (CGH) array was carried out using the Agilent 180K oligo-array (Amadid 023363, Agilent, Santa Clara, CA). The array-CGH detected 2 CNVs that were deemed not pathogenic, as both were inherited from the patient's healthy parents [arr [hg18] 9q32 (114,200,278-114,248,375) X3 mat, 17q23.3 (59,293,888-59,356,442) X1 pat].

**Figure 1:**
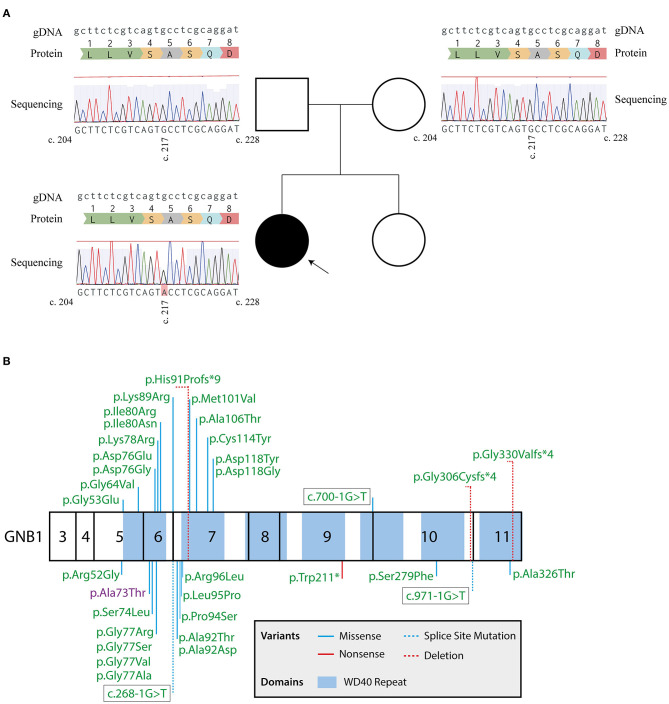
Sanger sequencing confirms a *de novo GNB1* variant. **(A)** Genogram of the proband with the respective results for *GNB1* sequencing of the patient and each parent. The c.217G>A heterozygous mutation was detected in the proband but not in the parents, indicating a *de novo* occurring variant. Results from Sanger sequencing were aligned with the genomic *GNB1* sequence (NG_047052.1) using the MAFFT 7 algorithm (20). The gDNA lane represents the genomic *GNB1* sequence; the protein lane represents the translation of amino acids from *GNB1* exon 6; the sequencing lane represents the results from Sanger sequencing of each patient, with a c.217G>A variant in the proband. **(B)** Mutation map of all *GNB1* variants implicated in GNB1 encephalopathy to the date of the study. Numbers inside each box represent the translated exons of *GNB1* (exons 1, 2, and 12 are not translated). Whenever possible, the mutation is represented by the change in protein structure. The variant detected in the patient is highlighted in purple.

Following this initial approach, a pre-defined panel of genes whose mutations are known to cause GDD/ID (Supplementary Table 1) was sequenced by massive parallel sequencing, using the SureSelect^XT^ Target Enrichment System (Agilent, Santa Clara, CA) for preparation of the DNA library, followed by sequencing using a MiSeq system (Illumina, San Diego, CA). Two heterozygote intronic variants were detected, one in the *SETD5* (NM_001282539.1; rs924035385) and one in the *TNIK* gene (NM_015028.3; rs192028546), both previously reported in the general population. Two heterozygote missense mutations were also detected, namely in the *HIVEP2* (NM_006734.3; c.5866G>A; p.Gly1956Arg) and *GNB1* (NM_001282539.1; c.217G>A; p.Ala73Thr) genes, with both genes having been implicated in autosomal dominant GDD. However, the *HIVEP2* variant is categorized as likely benign, with *in silico* predictions strongly supporting a neutral effect of the mutation in protein function (Condel, 0.317; SIFT, 0.630; Polyphen-2, 0.013; Massessor, −0.205). In contrast, the *GNB1* variant is predicted to be deleterious by several *in silico* tools (Condel, 0.558; SIFT, 0.000; Polyphen-2, 0.976; Massessor, 1.000). Moreover, and as previously described, *GNB1* is highly intolerant to genetic variation, with disruptions to protein structure leading to profound changes in its function (1). Finally, the *GNB1* variant was confirmed by Sanger sequencing in the patient and shown to occur *de novo*, since it was not detected in either parent (Figure 1A). Therefore, this novel *GNB1* variant can be categorized as pathogenic (as it fulfills 2 strong criteria for pathogenicity, PS2 and PS3) (21). Interestingly, this variant maps to exon 6, one of the two *GNB1* exons with mutational hotspots (Figure 1B).

**Figure 2:**
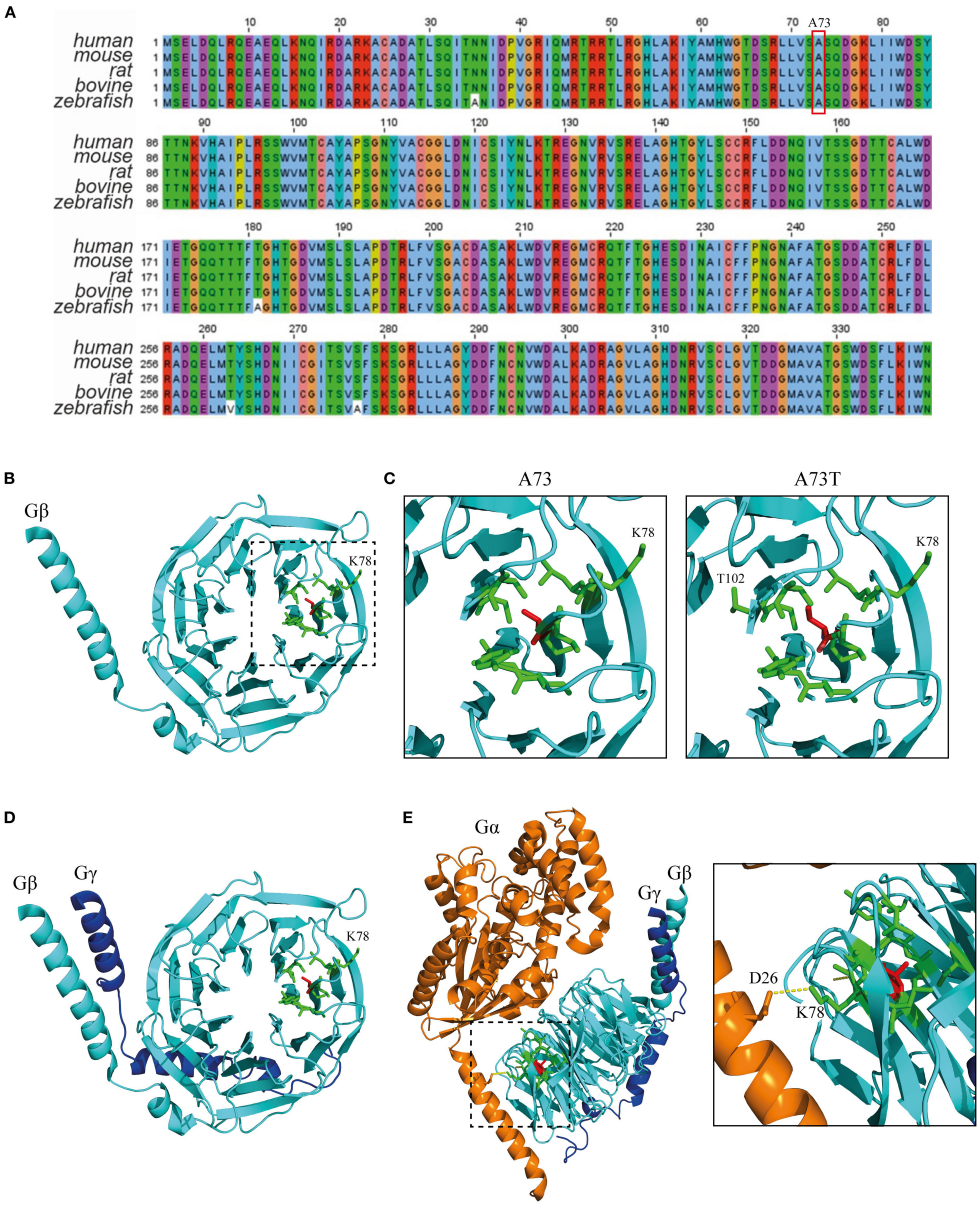
The A73T mutation might compromise a WD40 repeat from the WD40 domain of GNB1. **(A)** Sequence alignment of GNB1 protein from different species demonstrating that the amino acid sequence is almost unchanged across evolution. The alignment was performed using Jalview (Version 2) software (22). **(B)** Three-dimensional structure of GNB1 (pdb −1gp2, chain B). A73 (red sticks) is located in a core β-sheet from an WD repeat (boxed) that composes the circularized β-propeller WD40 domain of GNB1. Represented in green sticks are the residues that distance less than 4A of A73. **(C)** Three-dimensional structure detail of the WD repeat encompassing A73 residue (left) and simulated A73T mutagenesis (right). Residues that are within 4A distance that might be stabilized by A73 are represented as green sticks. Upon A73T mutagenesis, using the highest probable side chain orientations for threonine, an additional residue (T102) from an adjacent WD40 repeat appears within 4A distance, indicating that A73T might have an impact on this region. **(D)** Three-dimensional structure detail of the Gβγ dimer, showing that A73 is unlikely to be required for dimer formation. **(E)** Three-dimensional structure detail of the Gα*βγ* trimer. The inset represents the interaction between Gα D26 and Gβ K78 that is required for trimer assembly, which is potentially affected by the A73T mutation. Structural modeling was performed using PyMOL (23).

GNB1 (Uniprot: P62873) is an extremely well-conserved protein. In fact, the amino acid sequence is identical between humans and other mammals, such as mouse, rat and bovine, and differs only by 4 residues from zebrafish (Figure 2A). This demonstrates that almost no mutations were selected during evolution, indicating that any mutation could possibly have a dramatic structural impact with consequent loss of function. A73 is located in a core beta sheet from a WD40 repeat motif that is part of the circularized β-propeller WD40 domain of the GNB1 protein (Figure 2B). WD40 domains are mainly involved in the assembly of large molecular complexes and the WD motifs act as protein interaction scaffolds. A73 lies within 4A from 9 residues: I58, Y59, A60, S72, S74, K78, C79, V100 and C103 (Figure 2C, left). Substitution of the non-polar A73 for the polar hydroxyl residue threonine (A73T, Figure 2C, right) might locally impact the structure. It is particularly important for lysine 78, a residue whose mutation (K78R) was already identified as causal of GNB1 encephalopathy (1). Moreover, simulation of the A73T substitution, using the highest probable side chain orientations for threonine, revealed an additional residue (T102, Figure 2C, right) in the proximity (within 4A) that belongs to an adjacent WD40 motif. This indicates that any destabilization caused by the A73T substitution might have a broader impact on the WD40 domains of GNB1. Interestingly, while A73 is not involved in the formation of the Gβγ dimer (Figure 2D), K78 establishes a polar contact with D26 from Gα (Figure 2E), helping in the stabilization of the Gα N-terminal helix near the β-propeller domain of Gβ. A destabilization of this region might therefore impact the Gα-Gβγ interaction, with important functional consequences. Given the position of A73 within the WD40 motif and the functionality of the WD40 domain, a mutation in this residue could also impact G protein-effector interactions.

## Discussion

We have described a new case of GNB1 encephalopathy, namely a patient with profound GDD, ID and a complex behavioral disorder. Genetic testing uncovered a novel pathogenic *de novo GNB1* mutation which affects a different residue from all other described patients, with *in silico* predictions indicating that the abnormal protein is likely to be functionally compromised. Similarly to more than 50% of the described series (6), the presented patient displays developmental delay and ID. Less frequent manifestations that were also observed in the patient are behavioral issues (usually ADHD and ASD). Frequent GNB1 encephalopathy symptoms that were not observed are abnormal muscle tone, abnormal vision and epilepsy. Nonetheless, the sole finding that is common to 100% of patients is GDD.

This is the first described patient with a substitution on residue A73, which is encoded in exon 6 of the *GNB1* gene. This supports the hypothesis of the presence of a mutational hotspot spanning exons 6 and 7, as 88% of all missense mutations map to his region. Functional studies have shown that GPCR-dependent signal transduction that requires Gβ1 activity is decreased by all exon 6–7 mutations described to the date of the study, suggesting that this is a critical region for protein function (9). Nevertheless, mutations in other regions also disrupt protein function and cause a similar disorder. In fact, GNB1 has several WD40 domains that assemble to form a tridimensional β-propeller interface to which Gγ proteins bind, and an opposite β-barrel surface for Gα binding (24). Therefore, key residues that span most of the length of the protein have been implicated in these interactions, suggesting that mutations outside of the predicted hotspot can be equally damaging.

One of the effector interactions that could be destabilized by the A73T substitution is the interaction of GNB1 with the β-adrenergic receptor kinase 1, GRK2. GNB1 tyrosine 59 is important for GNB1:GRK2 interaction, since it stabilizes K663 and M664 from GRK2 (25). Any structural impact on Y59 caused by the A73T mutation could impact GNB1:GRK2 interaction. Interestingly, Y59 is also part of the interaction surface between GNB1 and phosducin, a protein involved in the regulation of visual phototransduction (26).

In conclusion, we have described a case of GNB1 encephalopathy with a *de novo* mutation which affects a residue that has not been previously implicated in disease. While other variants might contribute to the observed phenotype, it is extremely likely that the *GNB1* variant is the key alteration that caused disease. In fact, the patient presents with key aspects of the disorder and has a mutation that is very likely to disrupt protein function. The addition of new cases to the literature of this condition are important (27), not only to expand the understanding of this rare syndrome, but also in the hope of working toward potential treatments.

## Data Availability Statement

The original contributions presented in the study are included in the article/Supplementary Material. Further inquiries can be directed to the corresponding author/s.

## Ethics Statement

Written informed consent was obtained from the minor(s)' legal guardian/next of kin for the publication of any potentially identifiable images or data included in this article.

## Author Contributions

JDS: drafting/revision of the manuscript for content, including medical writing for content, major role in the acquisition of data, study concept or design, and analysis or interpretation of data. MDC and FL: drafting/revision of the manuscript for content, including medical writing for content, major role in the acquisition of data and analysis or interpretation of data. BA: drafting/revision of the manuscript for content, including medical writing for content and analysis or interpretation of data. PM and AT-C: drafting/revision of the manuscript for content, including medical writing for content, study concept or design, and analysis or interpretation of data. All authors contributed to the article and approved the submitted version.

## Funding

This work was funded by National funds, through the Foundation for Science and Technology (FCT) - project UIDB/50026/2020 and UIDP/50026/2020 and by the project NORTE-01-0145-FEDER-000039, supported by Norte Portugal Regional Operational Programme (NORTE 2020), under the PORTUGAL 2020 Partnership Agreement, through the European Regional Development Fund (ERDF).

## Conflict of Interest

The authors declare that the research was conducted in the absence of any commercial or financial relationships that could be construed as a potential conflict of interest.

## Publisher's Note

All claims expressed in this article are solely those of the authors and do not necessarily represent those of their affiliated organizations, or those of the publisher, the editors and the reviewers. Any product that may be evaluated in this article, or claim that may be made by its manufacturer, is not guaranteed or endorsed by the publisher.
