# Supplementary material for: Case Report: A Novel GNB1 Mutation Causes Global Developmental Delay With Intellectual Disability and Behavioral Disorders
Source: Front Neurol. 2021 Sep 27;12:735549. doi: 10.3389/fneur.2021.735549 (PMC8504539; doi:10.3389/fneur.2021.735549)
Supplement: Supplementary file 1 [file Table_1.docx]

**Supplemental Information**

**Supplemental Table 1.** Panel of genes that were sequenced by massive parallel sequencing in the patient.

| ACSL4 | CRADD | GDI1 | KIAA2022 | NONO | RPS6KA3 | TRAPPC9 |
| --- | --- | --- | --- | --- | --- | --- |
| ADAT3 | CRBN | GNB1 | KIF1A | NSDHL | SETBP1 | TRIP12 |
| ADNP | CTCF | GNB5 | KIF4A | NSUN2 | SETD5 | TSPAN7 |
| AFF2 | CTNNB1 | GPT2 | KIRREL3 | OPHN1 | SHROOM4 | TTI2 |
| AHDC1 | CUL4B | GRIA3 | KLHL15 | PACS1 | SLC16A2 | TUSC3 |
| ANK3 | DDX3X | GRIK2 | KMT5B | PAK3 | SLC6A17 | UBE2A |
| APIS2 | DEAF1 | GRIN1 | KPTN | PGAP1 | SLC9A6 | UPF3B |
| ARHGEF6 | DLG3 | GRIN2B | L1CAM | PGAP2 | SMARCA4 | USP27X |
| ARID1A | DOCK8 | HBA1 | LINS1 | PHF6 | SMARCB1 | USP9X |
| ARID1B | DPP6 | HCFC1 | LMAN2L | PHF8 | SMS | ZBTB18 |
| ARX | DYNC1H1 | HDAC8 | MAN1B1 | PIGG | SOX11 | ZC3H14 |
| ASH1L | DYRK1A | HERC2 | MBD5 | POGZ | SOX3 | ZDHHC15 |
| ATP6AP2 | EDC3 | HIVEP2 | MBOAT7 | PPP2R1A | SRPX2 | ZDHHC9 |
| ATRX | EEF1A2 | HNMT | MECP2 | PPP2R5D | ST3GAL3 | ZMYM3 |
| AUTS2 | EIF2S3 | HNRNPH2 | MED12 | PQBP1 | STAG1 | ZMYND11 |
| BRWD3 | ELP2 | HSD17B10 | MED23 | PRPS1 | SYNGAP1 | ZNF41 |
| CACNG2 | EPB41L1 | HUWE1 | MEF2C | PRSS12 | SYP | ZNF674 |
| CASK | EBXO31 | IGBP1 | METTL23 | PURA | TAF1 | ZNF711 |
| CC2D1A | FGD1 | IL1RAPL1 | MID2 | PUS3 | TAF13 | ZNF81 |
| CDH15 | FMN2 | IMPA1 | MRTO4 | RAB39B | TAF2 |  |
| CHAMP1 | FMR1 | IQSEC2 | MYT1L | RAB40AL | TBL1XR1 |  |
| CLCN4 | FRMPD4 | KAT6A | NAA15 | RBMX | TECR |  |
| CLIC2 | FTSJ1 | KDM5C | NDST1 | RLIM | THOC2 |  |
| COL4A3BP | GATAD2B | KIAA1033 | NLGN4X | RP2 | TNIK |  |
